# Attitudes and practices of community pharmacists and barriers to their participation in public health activities in Yemen: mind the gap

**DOI:** 10.1186/s12913-019-4133-y

**Published:** 2019-05-14

**Authors:** Seena A. Yousuf, Mohammed Alshakka, Wafa F. S. Badulla, Heyam Saad Ali, P. Ravi Shankar, Mohamed Izham Mohamed Ibrahim

**Affiliations:** 1 Social Medicine and Public Health Department, Faculty of Medicine and Health Sciences, Aden University, Aden, Yemen; 2 Section of Clinical Pharmacy, Faculty of Pharmacy, Aden University, Aden, Yemen; 3 Department of Analytical Chemistry, Faculty of Pharmacy, Aden University, Aden, Yemen; 4 Department of Pharmaceutics, Dubai Pharmacy College, Dubai, United Arab Emirates; 5 Department of Pharmacology, Health City University, Gros Islet, Saint Lucia; 6 Department of Clinical Pharmacy and Practice, College of Pharmacy, Qatar University, Doha, Qatar

**Keywords:** Community pharmacies, Cognitive services, Extended services, Health education, Health outcomes, Public health, Yemen

## Abstract

**Background:**

Patients in Yemen commonly visit community pharmacies to obtain consultation or treatment for common ailments. Community pharmacists have an opportunity to optimize medication use and improve patient outcomes. This study aimed to evaluate the attitudes and practices of community pharmacists regarding their participation in public health activities and barriers to their participation in these activities.

**Methods:**

This cross-sectional study was carried out among community pharmacists working in pharmacies located in urban areas of the Aden governorate of Yemen from March to June 2017 using a self-administered questionnaire. We selected pharmacies from a line list using proportional sampling according to the number of pharmacies in the urban areas of each district. The questionnaire contained four sections: demographic characteristics, attitudes, practices, and barriers encountered. Data were analyzed descriptively, and the Chi-square test was used for analyzing the association of variables (alpha = 0.05).

**Results:**

The questionnaire was distributed to 200 community pharmacists working in community pharmacies. Of the 200 respondents, 62% (*n* = 124) were male. Overall, the mean age (sd) was 30.0 years (8.6) with the number of years of work experience between 2 and 9.9 years (*n* = 158, 79%). On average, 62.3% of the pharmacists had a positive attitude toward participation in public health activities. Providing education to stop tobacco chewing, smoking, alcohol drinking and improve oral hygiene was an important activity of the community pharmacists. Blood pressure measurements (86%, *n* = 172) and glucose tests (45%, *n* = 90) were commonly conducted for clients. Lack of time (71%, *n* = 142) and lack of teamwork (70%, *n* = 140) were mentioned as common barriers to participation in public health activities.

**Conclusions:**

Community pharmacists had a positive attitude toward public health activities. Health education and routine health tests were important practices of the community pharmacists. Barriers need to be overcome to enable more active participation by community pharmacists in public health activities by consulting with all stakeholders, assessing the situation, considering alternatives and taking action.

**Electronic supplementary material:**

The online version of this article (10.1186/s12913-019-4133-y) contains supplementary material, which is available to authorized users.

## Background

Yemen is a low-income country whose health system suffers from lack of basic health care, poor facilities and lack of access to essential medicines [1–3]. Since 2015, sociopolitical conflict has affected all aspects of life, including health sector activities. Among the major medicine-related problems in the country are problems with prescribing and dispensing medication, improper use of medicine by consumers, problems with adverse drug reaction monitoring, and medical error [4–6]. Many patients view pharmacists in Yemen as drug sellers who only dispense and sell medicine [4]. Especially during the ongoing crisis, pharmacists in Yemen can play a critical role in supporting and maintaining public health. Pharmacists have an obligation, as well as opportunity to improve public health [7]. In Yemen, community pharmacists are only involved in the dispensing of medicine [4]. Most community pharmacists are not yet involved in providing public health services and health promotion activities. Despite the valuable role of community pharmacists, little research has examined their extended roles and tasks, beyond just dispensing and selling medicines. In Yemen, similar to many other low- and middle-income countries (LMICs), persons without pharmacy-related academic qualifications manage pharmacies and prescribe and dispense medicine.

Pharmacists are now considered important members of health care teams involved in decisions about drug use and adverse effects [8], particularly in community and hospital pharmacies [9, 10]. At the beginning of the twenty-first century, there were a number of calls to reform the pharmacy curricula to enable future pharmacists to meet their new roles and responsibilities [11]. These new roles necessitate improving clinical knowledge and skills toward patients [12, 13] and communicating with patients and other health care workers [14, 15].

Pharmacists provide a critical service to society and interact with people from diverse backgrounds. Due to the easy access and friendly approach of pharmacists, community pharmacies are among the first place patients visit to obtain consultation or treatment for their common ailments [16]. Moreover, pharmacists have an opportunity to participate in public health strategies, assist in decision-making and support public health efforts.

Public health has been defined as “what we as a society do to assure the conditions in which people live can be healthy” [17]. In contrast to medicine, public health initiatives emphasize the prevention of disease and consider the health needs of the population as a whole [18].

Community pharmacists have a role and responsibility to optimize medication use and improve patient outcomes and quality of life. Pharmacists are expected to possess a high level of personal skills to enable them to provide a good standard of practice and care and to deal with patients as individuals and respect their dignity. In dealing with other health care professionals, pharmacists must also follow standards of professional practice and establish a good working relationship with colleagues.

Ease of access to community pharmacists makes them the first choice to obtain health care for many patients [19]. Thus, the involvement of public health pharmacists in the health care system has been expanding in the last decade [16]. Hence, it is important to adopt suitable strategies to improve the participation of community pharmacists in public health activities. These improvements can only be carried out if there is continuous evaluation of the services provided at these pharmacies. On the other hand, healthcare providers in general and pharmacists in particular face a number of obstacles to providing good quality patient care.

Community pharmacists in Yemen have an important social obligation. They should be able to reach the community and provide extended services (i.e., cognitive services) during the state crisis. To date, there is little information in Yemen on community pharmacists’ level of contribution as public health practitioners. Thus, this study was carried out to explore the attitudes and practices of community pharmacists regarding their participation in public health activities and the barriers to their participation in these activities in the Aden governorate of Yemen.

## Methods

### Study design

An observational cross-sectional study was carried out from March to June 2017 among community pharmacists. The study used a validated self-administered questionnaire.

### Study site

Yemen has 20 governorates subdivided into districts. The study was carried out in Aden, one of the governorates of Yemen. Aden, which is the capital city of Yemen is one of the districts in the governorate. Other districts include Seera, Al Muaala, AlTawahi, Khormakser, Al Mansoora, Alshaikh-Othman, Dar-saad, and Alburaika, covering 7000 km^2^ with a population of 634,710. The study covered only the urban areas.

### Study population and sampling

The study population consisted of community pharmacists working at registered individually owned community pharmacies operating in the Aden governorate. Of the 468 community pharmacies on the list of pharmacies obtained from the Health Office of Aden, we estimated a sample size of 200 individuals based on a formula previously used for survey research [20]. The number of pharmacists from each district was proportional to the number of pharmacies in the district. Based on the geographic distribution of the pharmacies, we chose pharmacists using convenience sampling. If a pharmacist refused to participate or did not return the questionnaire within the required timeframe, we replaced the pharmacist with another from a different pharmacy in the same geographic area.

### Data collection and management

Data were collected using a self-administered questionnaire in the Arabic language that was developed through a review of relevant literature and expert opinion. The questionnaire had four sections (refer to Additional file 1). The first section explored demographic characteristics and practice information of the community pharmacists. The second section studied the attitudes of the community pharmacists toward public health activities and education using a five-point Likert scale; scores below the midpoint of 3 were considered to be positive responses. The ‘disagree’ and ‘strongly disagree’ responses on attitudes of community pharmacists toward public health activities were combined and calculated as the mean scores (sd) and the percentage of positive responses. As the statements were negatively worded, scores less than the midpoint of 3 indicated positive responses. The third section examined the practice (i.e., activities) of the community pharmacists using a five-point Likert scale; scores above the midpoint of 3 were considered to be positive responses. In addition, the medical tests that the community pharmacists commonly carried out in the pharmacy were investigated. The fourth section focused on barriers faced by the community pharmacists with regard to participation in public health activities.

A pilot study was performed among 40 pharmacists who were not included in the final study, and the internal consistency (i.e., Cronbach’s alpha) of the questionnaire was 0.748. The questionnaire was distributed by a researcher and trained pharmacist who instructed the community pharmacists to complete the survey within three days. The pharmacies were visited again after 3 days, and the researchers collected the completed questionnaires. The pharmacists were given another 48 h if the survey form still had not been completed. The head of the research team then checked the completeness of the self-administered questionnaires.

### Data analysis

Statistical Package for Social Sciences (SPSS) version 15 (SPSS Inc. Released 2007. SPSS for Windows, Version 15.0. Chicago, SPSS Inc.) was used for data entry and analysis. The frequency of various responses was noted. The mean (sd), frequency distribution, and percentage of the variables were calculated. The Chi-square test determined whether variables such as age, gender, years of experience, and educational level influenced the questionnaire scores at a significance level of 0.05.

## Results

The internal consistency (i.e., Cronbach’s alpha) of the final questionnaire was 0.717. The majority of the respondents were male (*n* = 124, 62%) with an overall average age (mean ± sd) of 30.0 ± 8.6 years. The dominant age group was 20–29 years old (*n* = 132, 66%), followed by the 30–39-year-old age group (*n* = 44, 22%). With regard to the educational level of the respondents, 51% (*n* = 102) of pharmacists had a diploma, and 49% (*n* = 98) had a bachelor’s degree. Of the respondents, 87% had less than 10 years (median (IQR) = 5.0 (2.4–7.0)) of work experience. The distribution of pharmacists/pharmacies by location showed that 40% of the community pharmacists were from the Al-Mansoora district, followed by 25% from the AlShikh-Othman district (Table 1).

**Table 1:** Demographic characteristics of community pharmacists and pharmacies

Characteristics	Frequency (%)
Gender	
Male	124 (62.0)
Female	76 (38.0)
Age group (years)	
20–29	132 (66.0)
30–39	44 (22.0)
40–49	10 (5.0)
50–59	14 (7.0)
Educational level	
Bachelor in Pharmacy (BPharm)	98 (49.0)
Diploma in Pharmacy	102 (51.0)
Years of experience	
Less than 2	16 (8.0)
2–9.9	158 (79.0)
10–19.9	8 (4.0)
20 & more	18 (9.0)
Number of pharmacies in each directorate/district	
Al-Buraika	10 (5.0)
Al-Manssora	80 (40.0)
Al-Mualaa	10 (5.0)
Dar-Saad	10 (5.0)
Khormaksar	10 (5.0)
Seera	20 (10.0)
AlShikh-Othman	50 (25.0)
AlTawahi	10 (5.0)
Number of clients daily	
Less than 30	40 (20.0)
30 - < 59	106 (53.0)
60 - < 89	22 (11.0)
90 & more	32 (16.0)
Total	200 (100)

Relating to attitudes toward participation to public health activities, on average, 62.3% of the pharmacists had positive responses to statements (Table 2). The results indicated only one negative attitude response to the statement - ‘Public health activities belong only to health centers’ (3.44 ± 1.23; 47%). The other statements showed that the community pharmacists were positive (reversed statements).

**Table 2:** Community pharmacists’ attitudes towards public health activities

Statement	Mean (SD)	Positive responses (%)
Pharmacists should not be involved in public health activities	1.51 (0.99)	92
People will not accept my participation in public health activities	1.98 (0.83)	83
I am not ready to be involved with public health activities.	2.02 (1.08)	75
It is not important for pharmacists to practice health promotion activities.	2.13 (1.03)	72
Health education only to problems related to drugs should be provided.	2.59 (1.27)	66
I am not interested in public health activities as it is the work of doctors and nurses	2.27 (1.01)	64
I don’t have enough knowledge to advice patients on health promotion and disease prevention.	2.23 (1.17)	63
Public health activities belong to health centers	3.44 (1.23)	47
I do not have the time to educate patients on health issues.	2.83 (0.95)	41
Other health workers do not allow pharmacists to carry out activities related to public health.	2.92 (0.66)	20

The Chi-square test results showed that the questionnaire scores were significantly different with regard to age (*p* < 0.001), gender (*p* < 0.001), years of experience (*p* < 0.001), and educational level (p < 0.001) (Table 2).

Community pharmacists noted that the most sought after service that they delivered was education and information related to oral health (3.93 ± 0.91, 96%). Moreover, they also provided information on how to stop smoking cigarettes (4.30 ± 0.98, 91%) and chamna (a form of tobacco) (4.50 ± 0.90, 95%), chewing khat (the leaves of an Arabian shrub, which are chewed or drunk as a stimulant) (4.14 ± 1.02, 91%), and drinking alcohol (4.40 ± 1.07, 92%), and on immunodeficiency syndrome (4.21 ± 1.05, 88%) and how to follow a proper nutrition regime (4.49 ± 0.88, 90%). (Table 3).

**Table 3:** Community pharmacists’ public health activities

Statement	Mean (SD)	Positive responses (%)
Oral health	3.93 (0.91)	96
Education to stop Chamma	4.50 (0.90)	95
Education to stop alcohol drinking	4.40 (1.07)	92
Education to stop Khat chewing	4.14 (1.02)	91
Education to stop smoking	4.30 (0.98)	91
Education to follow healthy nutrition regime	4.49 (0.88)	90
Information about acquired immunodeficiency syndrome	4.21 (1.05)	88
Information about contraceptive methods	3.92 (0.94)	82
Information about cancer	3.89 (1.02)	81
Unused or expired drugs	3.90 (1.01)	76
Medical instruments	3.65 (1.19)	70
Education to follow a healthy lifestyle	3.73 (1.04)	69
Weight reduction	3.39 (1.13)	56
Cholesterol balance	3.24 (0.99)	47

**Fig. 1:**
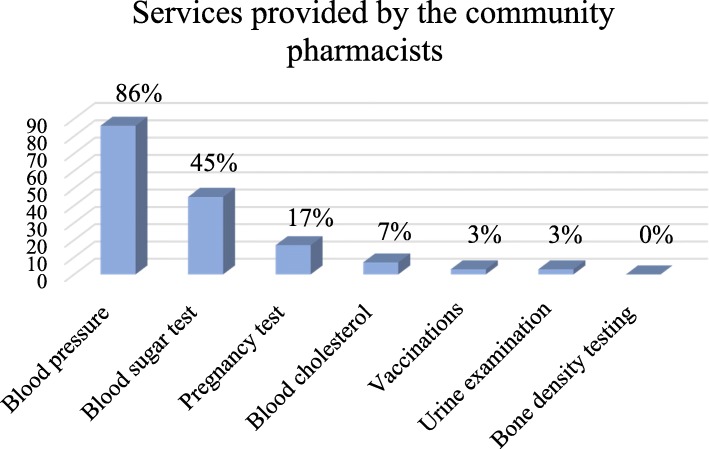
Services provided by the community pharmacists

Of the community pharmacists who mentioned that they provided health-related diagnostic tests for clients, 86% (*n* = 172) performed blood pressure measurements, 45% (*n* = 90) conducted blood glucose tests, 17% (*n* = 34) performed pregnancy tests, 7% (*n* = 14) conducted blood cholesterol tests and 3% (*n* = 6) did urine examinations. Vaccinations were provided by 3% (n = 6) of the community pharmacists (Fig. 1).

With regard to participation in public health activities, 63% (*n* = 126) of community pharmacists felt that they could contribute to educational activities related to public health, such as health advancement and disease prevention, if given proper training. Among the barriers they cited were lack of time (71%, *n* = 142), lack of teamwork (70%, *n* = 140) and lack of outside funds (58%, *n* = 116) (Table 4).

**Table 4:** Barriers toward involvement of community pharmacists in public health activities

Statements	*N* (%)
Lack of time	142 (71.0)
Lack of the concept of teamwork	140 (70.0)
Lack of outside funds to assist pharmacists	116 (58.0)
Lack of training	112 (56.0)
Lack of financial capital to implement changes	108 (54.0)
Lack of official recognition of public health activities	108 (54.0)
Lack of the concept of public health activities	98 (49.0)
Lack of specialty and experience	86 (43.0)

## Discussion

This study evaluated the attitudes and practices of community pharmacists regarding their participation in public health activities and the barriers preventing their participation in these activities in the Aden governorate of Yemen. The community pharmacists had a positive attitude to involvement in public health activities. They were engaged in educational activities related to personal life style practices, along with health monitoring tests. Barriers noted were lack of time to consult with clients and lack of teamwork with their colleagues.

Almost two-thirds of community pharmacists felt that people will accept pharmacists’ participation in public health activities, and they were ready to be involved with these activities. Armstrong et al. [21] stressed that community pharmacists can play an important role in public health and they need to be competent in different areas of practice. There are three domains that community pharmacists can be involved in i.e. health protection and prevention, health and social care, and health improvement [22]. Walker referred to these practices as ‘pharmaceutical public health’ [23]. The number of skilled and educated community pharmacists available for counseling patients about specific issues related to the prescribed medication, and reporting common adverse effects, was low according to a previous study conducted in Aden, Yemen [24].

The feedback obtained from the majority of the community pharmacists showed positive attitude toward involvement in public health activities and recognition of its vital contribution. The results of a study by Offu and coworkers was similar to our study and demonstrated positive attitude among the community pharmacists towards public health practices even though the knowledge and practice level were not satisfactory [25]. Other studies and a systematic review showed a similar situation [26–28]. Many community pharmacists were willing to be a part of public health activities, having knowledge about the importance of practicing health promotion activities and they were interested in supporting public health activities as a part of the health care team. A study in Ethiopia indicated a low level of participation of community pharmacists in public health activities [29]. In this study, fourth-fifth of respondents claimed that the other health workers do not allow pharmacists to carry out public health activities. Hence, good communication and cooperation between different health care professionals should be established [30]. However, the public acceptance of the participation of community pharmacists in public health activities is perceived as positive. All these findings suggest that there is an acceptance from both the community pharmacists and patients toward changing from the traditional role of dispensing medication to more effective involvement in health care activities.

The community pharmacists felt it was important for pharmacists to practice health promotion activities. They also had a positive opinion regarding providing health education beyond drug-related issues, and believed that public health activities are not just the responsibility of doctors and nurses. They also were of the opinion that community pharmacists have enough knowledge to advice patients on health promotion and disease prevention. They claimed that they have been practicing various public health activities such as oral health; educating the clients on matters related to chamna, alcohol drinking, khat chewing, smoking, health nutrition, healthy lifestyle; providing information on immunodeficiency syndrome; providing information on contraceptives, cancer drugs, matters related to expired and unused medicines, the use of medical devices, weight reduction and cholesterol. Among the medical tests commonly conducted by community pharmacists were blood pressure, glucose and pregnancy tests. The educational activities conducted by community pharmacists for their patients as part of the public health activities were consistent with the activities provided by the community pharmacists in other low- and middle-income countries [31]. Agomo et al. reported that the literature revealed that services such as smoking cessation, health promotion, provision of hormonal contraceptives and preventive activities provided by pharmacists have benefited the community [32]. Community pharmacists carried out medical tests such as blood pressure measurement and blood glucose test. By offering these screening tests, the community pharmacists may assist patients with diagnosis and therapy. The provided medical tests were similar to the services offered by community pharmacists in Dhaka, Bangladesh [31].

The community pharmacists stated several barriers hindering their involvement in public health activities. Among them were lack of time, lack of understanding of the concept of teamwork among the different healthcare professionals, lack of understanding of the concept of public health, shortage of funds and capital to implement improvements, lack of training, recognition, specialty and experience. The last part of the current study related to the obstacles and difficulties facing the community pharmacists with regard to effective contribution to public health activities. The major obstacles reported in the current survey were lack of time, and of the concept of teamwork followed by shortage of external funds and lack of official recognition of participation in public health activities. Comparable barriers have also been reported in some developing countries like Ethiopia and southeast Nigeria [29, 32]. The reported barriers can be partly overcome if there is willingness to provide free public health services rather than charging services to improve business profitability by the community pharmacists. In addition, the healthcare professionals must embrace the concept of collaborative care i.e. teamwork between physician and pharmacist. Public health activities can be performed and enhanced by working together. There are key areas such as interprofessional collaborative care, working with and for communities that community pharmacists should consider as initial steps for their involvement in public health activities [33].

Even though the study provided some insights on the pharmacy public health activities in Yemen, it has a few limitations. The study was conducted with a small sample and only part of the registered community pharmacists; thus, the findings may not represent the actual perspective of community pharmacists in the entire country. Furthermore, the study was only conducted in one of the governorates of Yemen due to the war and safety issue. Thirdly, self-perception of community pharmacists i.e. relying on self-reported information may contribute to bias.

The authors wish to recommend the following actions. First, from the research angle, future studies need to be carried out on a larger geographical area and sample size, as well as using different methods such as simulated patient method. There should be more researches that study the current situation, identify possible problems and suggest solutions. Secondly, from the policy and practice viewpoints, the basic core services provided by pharmacists to the public should be strengthened especially when state is at crisis [34, 35]. In order to improve the provision of pharmaceutical care, Farris and Schopflocher recommended accessibility of qualified and cooperative staff for dispensing activities [36]. Pharmacists need to be well-trained, competent and confident in order to have positive outcome [26]. Offering specific training is a necessary requirement to boost community pharmacists’ involvement in the health care services. Community pharmacists should have time to participate in continuing professional education to upgrade the level of the provided services. However, the quality and impact of the services on the individuals and public are not known; thus, it need to be investigated. Evaluation of the community pharmacist’s contribution must be carried out to strengthen their contribution to public health activities. Various stakeholders should collaborate for improving the services and enhancing the competency of the community pharmacists. Community pharmacists must be accepted as an important member of the health care team. Policymakers should develop appropriate policies to improve the involvement of community pharmacists in public health activities in Yemen.

## Conclusions

In Yemen, as in many LMICs, the role and contribution of community pharmacists is not well appreciated even though they are located in a unique position. This study highlighted the gaps with regard to community pharmacists’ roles, and contributions to public health services, especially during the state crisis. Health facilities in Yemen are badly affected and in need of humanitarian support. Thus, community pharmacists need to be increasingly involved in public health activities. The study concluded that the community pharmacists had positive attitudes toward public health activities, had been practicing public health activities to a certain extent, but had identified barriers that prevented their effective contribution as public health practitioners.

## Additional file

Additional file 1: Questionnaire on attitudes, practices and barriers of community pharmacists to public health activities in Yemen. (PDF 180 kb)

## Abbreviation

LMICs: Low- and Middle-Income Countries
