# Supplementary material for: Attitudes and practices of community pharmacists and barriers to their participation in public health activities in Yemen: mind the gap
Source: BMC Health Serv Res. 2019 May 14;19:304. doi: 10.1186/s12913-019-4133-y (PMC6515656; doi:10.1186/s12913-019-4133-y)
Supplement: Supplementary file 1 — Questionnaire on attitudes, practices and barriers of community pharmacists to public health activities in Yemen. (PDF 180 kb) [file 12913_2019_4133_MOESM1_ESM.pdf]

بسم الله الرحمن الرحيم

جامعة عدن

قسم الصحة العامة و طب المجتمع, كلية الطب والعلوم الصحية [1]

شعبة الصيدلة السريرية, كلية الصيدلة [2]

أعزائي وعزيزاتي / الصيدلانيين

السلام عليكم ورحمة الله وبركاته ,,,

يرجى من سيادتكم التعاون معنا والمشاركة الطوعية في الدراسة التالية , " ادراك صيادلة المجتمع لمشاركتهم في انشطه الصحة العامة في المدينة والريف في اليمن." وتطبق في مديريات محافظتي عدن و لحج وتهدف الى استطلاع أراء عينة من الصيادلة و اخذ بعض المقاييس التي تخدم موضوع الدراسة المبين أعلاه .

تتكون الاستبانة التي بين يديكم من ستة محاور تتضمن المواضيع التالية:

المحور الأول الخصائص الديموغرافية, المحور الثاني المواقف , المحور الثالث الأنشطة الصحية التي يجب أن يقوم بها الصيدلي , المحور الرابع الفحوصات التي يقوم بها الصيدلي في الصيدلية , المحور الخامس المعوقات والمشاكل التي تحد من قيام الصيدلي بالأنشطة الصحية والترويجية في الصيدلية و المحور السادس خاص بالأنشطة الصحية في الأرياف .

مع العلم بأن المعلومات التي ستجمع في هذه الاستبانة لن تستخدم إلا لأغراض البحث العلمي .

أرجو منكم الإجابة على الأسئلة بكل صدق وموضوعية كما عهدنا منكم لضمان نتائج مثمرة للبحث

شاكرين حسن تعاونكم

وتقبلوا كل التقدير والاحترام.

الباحثان

د. سيناء يوسف [1]

د. محمد الشقاع [2]

استبيان باللغة العربية

<u>ادراك صيادلة المجتمع لمشاركتهم فى انشطه الصحة العامة فى المدينة والريف فى اليمن.</u>

<u>المحور الأول:</u> الخصائص الديموغرافية

1. الجنس ذكر ☐ أنثى ☐
2. العمر بالسنوات ☐ سنة
3. الخبرة في العمل بالسنوات ☐ شهر ☐ سنة
4. المؤهل الدراسي:

فني صيدلة ☐ بكالوريوس صيدلة ☐ ماجستير صيدلة ☐ دكتوراه ☐

5. عدد الأشخاص المترددين إلى صيدليتك في اليوم تقريبا ☐ شخصا
6. مدينة ☐ ريف ☐

المحور الثاني: المواقف

الرقم	العبارة	1	2	3	4	5
1-	ليس لدي كصيدلي دراية كافية بتقديم النصح للمرضى بشأن التعزيز والترويج للصحة والوقاية من الأمراض.					
2-	لا ينبغي أن يكون للصيادلة اسهام كبير في الصحة العامة.					
3-	لا يرغب الصيدلي في الترويج للصحة لأنه عمل الطبيب والممرض.					
4-	ليس لدى الصيدلي الوقت الكافي للترويج الصحي.					
5-	المهنيين الصحيين الآخرين لا يقبلوا دور الصيدلي في الترويج الصحي وتعزيز الصحة.					
6-	ليس مهما أن يمارس صيدلي المجتمع الترويج للأنشطة الصحية.					
7-	أنا كصيدلي غير مستعد للمشاركة في الحملات الصحية وأنشطة الترويج للصحة العامة.					
8-	أنشطة الصحة العامة يجب أن تقتصر على المراكز الصحية.					
9-	عامة الناس لا تتقبل مشاركة الصيادلة في الأنشطة الصحية في المجتمع.					
10-	التثقيف الصحي في الصيدلية يجب أن يقتصر على المشاكل المتعلقة بالدواء.					

1. غير موافق بشدة 2. غير موافق 3. لست متأكدا 4. أوافق 5. أوافق بشدة

المحور الثالث: الأنشطة الصحية التي يجب أن يقوم بها الصيدلي

الرقم	العبارة	1	2	3	4	5
-1	تثقيف المرضى بشأن الاقلاع عن التدخين.					
-2	تثقيف المرضى بشأن الاقلاع عن تعاطي القات.					
-3	تثقيف المرضى بشأن الاقلاع عن تعاطي الشمة.					
-4	تثقيف المرضى بشأن الاقلاع عن تعاطي الكحول.					
-5	تثقيف المرضى بشأن اتباع نظام غدائي , مثلا لمرضى السكر أو مرضى ارتفاع ضغط الدم .					
-6	ممارسة خاصة لبعض المرضى.					
-7	صيانة الكوليسترول العادي في الدم.					
-8	السمنة وتخفيف الوزن.					
-9	معلومات عن صحة الأسنان أو صحة الفم.					
-10	معلومات عن السرطان.					
-11	وسائل منع الحمل والأدوية.					
-12	الإيدز والوقاية منه.					
-13	الأدوية غير المستعملة والنفايات الدوائية.					
-14	الأجهزة الطبية والصحية , مثلا لقياس ضغط الدم ومستوى السكر في الدم.					

1. غير متورط للغاية 2. غير متورط 3. ليس من المؤكد 4. تشارك 5. تشارك جدا

المحور الرابع: الفحوصات التي يقوم بها الصيدلي في الصيدلية

الرقم	السؤال	نعم	لا	أحيانا
-1	فحص السكر في الدم			
-2	فحص الكوليسترول في الدم			
-3	قياس ضغط الدم			
-4	فحص الحمل			
-5	القيام بإجراء اللقاحات			
-6	فحص كثافه العظام			
-7	فحص البول			

المحور الخامس: المعوقات والمشاكل التي تحد من قيام الصيدلي بالأنشطة الصحية والترويجية في الصيدلية.

1. قلة الوقت.
2. قلة الخصوصية.
3. نقص رأس المال المالي لتنفيذ التغييرات في مهام الصيدلي.
4. عدم التعويض المالي من قبل الجهات المعنية.
5. نقص التدريب.
6. عدم الفهم الكامل بالأنشطة الصحية.
7. عدم الاعتراف الرسمي بالأنشطة الترويجية للصحة.
8. عدم وجود مفهوم لتكامل فريق العمل مع أعضاء الرعاية الصحية الأخرى.

شكرا جزيلا على تعاونكم

الباحثان

Aden University

1) Department of Public Health and Community Medicine, Faculty of Medicine and Health Sciences.
2) Department of Clinical Pharmacy, Faculty of Pharmacy.

Dear Sir and Madam / Pharmacists,

Peace, mercy and blessings of God,

You are kindly requested to cooperate with us and participate voluntarily in the following study, " Attitudes, practices and barriers of community pharmacists to public health activities in Yemen ". The study will be conducted in Aden Governorate and aims to survey the views of a sample of pharmacists and obtain information about the subject mentioned above.

The questionnaire in your hand consists of five dimensions:

The first dimension is, the demographic characteristics, the second dimension is, the attitude, the third dimension is the health activities to be carried out by the pharmacist, the fourth dimension is, the pharmacist's tests in the pharmacy, and the fifth dimension is the obstacles and the problems that limit the pharmacist's health and promotional activities in the pharmacy,

The information collected in this questionnaire will only be used for the purposes of the study. All individual data will be kept confidential and only the group data will be used for publication purposes.

I ask you to answer questions as honestly and objectively as you can to obtain objective information about the study subject

Thank you for your cooperation

With all respect

The researchers

Attitudes, practices and barriers of community pharmacists to public health activities in Yemen

First dimension: Demographic characteristics

1. Gender Male ☐ Female ☐
2. Age in years ☐
3. Work experience in years ☐
4. Educational qualification
   - Diploma of Pharm. Tech. ☐ - Bachelor of Pharmacy ☐
   - ------------------------------ ☐
5. Number of customers seen per day ☐

Second dimension: Attitudes

No.	Statement	1	2	3	4	5
1	I don't have enough knowledge to advice patients on health promotion and disease prevention.					
2	Pharmacists should not be involved in public health activities					
3	I am not interested in public health activities as it is the work of doctors and nurses					
4	I do not have the time to educate patients on health issues.					
5	Other health workers do not allow pharmacists to carry out activities related to public health.					
6	It is not important for pharmacists to practice health promotion activities.					
7	I am not ready to be involved with public health activities.					
8	Public health activities belong to health centers					
9	People will not accept my participation in public health activities.					

10	I can provide health education only for problems related to medicines.					

1. Not agree at all 2. Not agree 3. I don't know 4. Agree 5. Strongly agree.

Third dimension: Health activities carried out by community pharmacist

No.	Statement	1	2	3	4	5
1	I provide education to stop smoking					
2	I provide education to stop Qhat chewing					
3	I provide education to stop Chamma use.					
4	I provide education to stop alcohol drinking					
5	I provide education to follow healthy nutrition regime					
6	I provide education to follow special life style					
7	I provide education to maintain cholesterol balance					
8	I provide education to decrease weight					
9	I provide information about oral health					
10	I provide education about cancer					
11	I provide information about contraceptive methods					
12	I provide information about acquired immunodeficiency syndrome (AIDS)					
13	I provide education about unusable or expired drugs					
14	I provide education about medical instruments					

1. Not agree at all 2. Not agree 3. I don't know 4. Agree 5. Strongly agree.

Fourth dimension: Tests conducted by the pharmacist in the pharmacy

No.	Investigation	Yes	No	Sometimes
1	Blood pressure measurement			
2	Blood sugar test			
3	Pregnancy test			
4	Blood cholesterol test			
5	Vaccinations			
6	Bone density testing			
7	Urine examination			

Fifth dimension: Obstacles and problems that limit the pharmacist's health and promotional activities in the pharmacy.

1. Lack of time. ☐
2. Lack of privacy. ☐
3. Lack of financial capital to implement changes in the pharmacist's duties. ☐
4. Lack of financial compensation by the concerned authorities. ☐
5. Lack of training. ☐
6. Lack of full understanding of health activities. ☐
7. Lack of formal recognition of promotional activities of health ☐
8. Lack of concept of team work among other health care members ☐

Thank you very much for completing the questionnaire and for your cooperation

The researchers
